# Renal Manifestations of IgG4-Related Disease: A Concise Review

**DOI:** 10.1155/2024/4421589

**Published:** 2024-06-24

**Authors:** Shahrukh T. Towheed, Wayel Zanjir, Kevin Yi Mi Ren, Jocelyn Garland, Marie Clements-Baker

**Affiliations:** Queen's University, Kingston, ON, Canada

## Abstract

IgG4-related disease (IgG4-RD) is an immune-mediated disorder marked by fibro-inflammatory masses that can infiltrate multiple organ systems. Due to its relatively recent discovery and limited understanding of its pathophysiology, IgG4-related disease may be difficult to recognize and is consequently potentially underdiagnosed. Renal involvement is becoming regarded as one of the key features of this disease. To date, the most well-recognized renal complication of IgG4-related disease is tubulointerstitial nephritis, but membranous glomerulonephritis, renal masses, and retroperitoneal fibrosis have also been reported. This concise review has two objectives. First, it will briefly encapsulate the history, epidemiology, and presentation of IgG4-related disease. Second, it will examine the reported renal manifestations of IgG4-related disease, exploring the relevant histology, imaging, clinical features, and treatment considerations. This synthesis will be highly relevant for nephrologists, rheumatologists, general internists, and renal pathologists to raise awareness and help improve early recognition of IgG4-related kidney disease (IgG4-RKD).

## 1. Introduction

Immunoglobulin G4-related disease (IgG4-RD) is a systemic immune-mediated condition characterized by dense lymphoplasmacytic infiltrates containing increased numbers of IgG4-secreting plasma cells. Affected organs frequently develop mass-like lesions that display a classic pattern of storiform fibrosis on histologic examination [1]. However, only recently has IgG4-RD become recognized as a unified clinical entity. Isolated organ manifestations of IgG4-RD have been reported separately for over 100 years [1, 2]. The disease was first described in the early 2000s affecting the pancreas as lymphoplasmacytic sclerosing pancreatitis (now known as Type 1 autoimmune pancreatitis) [3]. Since then, several additional syndromes have become recognized as IgG4-RD manifestations. In the head and neck, characteristic lymphocytic infiltration of the lacrimal, parotid, and salivary glands is known as Mikulicz's syndrome [4–6]. Characteristic involvement of the submandibular gland and thyroid gland are recognized as Kuttner's tumour [7] and Riedel's thyroiditis, respectively [8–10]. Inflammatory orbital disease (formerly orbital pseudotumour) is a recognized manifestation as well [11]. In the central nervous system, IgG4-related pituitary gland inflammation (hypophysitis) and pachymeningitis have been reported [12–14]. Thoracic involvement may include pericarditis [15], pleuritis [16], pulmonary pseudotumours [17], and mediastinitis [18]. Aortitis and periaortitis have also been documented [19, 20]. In the abdomen, hepatopathy and sclerosing mesenteritis are known manifestations [21, 22], and IgG4-related sclerosing cholangitis is now regarded as an independent entity from primary sclerosing cholangitis [23, 24]. Urinary and reproductive organ involvement may include ovarian, testicular, scrotal, and prostate gland infiltration [25, 26]. Finally, notable skin involvements may include cutaneous plasmacytosis and angio-lymphoid hyperplasia with eosinophilia [27]. Most certainly, the wide variety of possible organ manifestations and the many “faces” of this disease frequently make diagnosis complex and challenging [28, 29].

**Figure 1:**
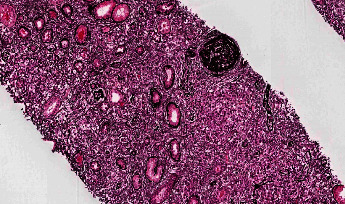
Storiform fibrosis highlighted on silver stain. Reproduced with permission from PathologyOutlines.com.

**Figure 2:**
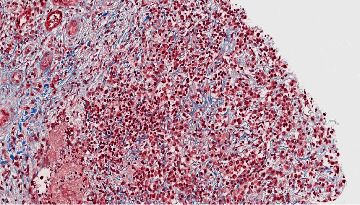
Storiform fibrosis highlighted on trichrome stain. Reproduced with permission from PathologyOutlines.com.

Renal involvement is now regarded as one of the key features of this disease. In 2004, the first reports of an association between Type 1 autoimmune pancreatitis and renal dysfunction were identified [30, 31]. Since that time, renal dysfunction has also been associated with Mikulicz's syndrome [32], IgG4-related hepatic involvement [33], and other extra-renal IgG4-RD syndromes [34, 35]. In these initial reports, renal involvement is manifested as tubulointerstitial nephritis, which to date is still the most well-recognized renal manifestation of IgG4-RD [34, 36]. However, glomerular involvement has also been described—membranous glomerulonephritis is the primary glomerular injury pattern noted in the literature [37, 38]. Additional described manifestations include renal masses [39, 40] and retroperitoneal fibrosis that can secondarily affect the renal system [41, 42]. The diversity of potential manifestations in the kidney has led to more encompassing terminology entitled IgG4-related kidney disease (IgG4-RKD) [43]. The first set of IgG4-RKD diagnostic criteria was proposed in 2011 by Kawano and colleagues [43]. An updated version by Saeki and colleagues was proposed in 2020 and is enclosed in Table 1 [44]. In practice, tissue examination by means of renal biopsy in combination with clinical, laboratory, and imaging features is used to confirm the diagnosis of IgG4-RKD. Storiform fibrosis under the microscope is depicted in Figures 1 and 2.

## 2. Presentation

IgG4-RD is an uncommon disorder. There are few objective data available regarding international prevalence and incidence. IgG4-RD is most extensively studied in Japan, where national estimates of prevalence and incidence range between 8,000 and 10,000 affected persons and 0.28–1.08/100,000 population, respectively [45, 46]. However, IgG4-RD has been reported across racial and ethnic groups, and it is likely underrecognized in other parts of the world [47]. The peak age of affected individuals is between 50 and 70 years old with a male predominance [45, 47]. Among patients with IgG4-RKD, it appears that renal involvement is typically accompanied by involvement at other extra-renal sites [34, 43, 48].

The pathophysiology of IgG4-RD remains elusive and is not fully understood. Similar to many autoimmune disorders, genetic factors, autoimmunity, and allergy each may contribute uniquely to its pathogenesis. It is currently hypothesized that type-2 helper T-cells and regulatory T-cell cytokines possibly drive disease pathogenesis, while the IgG4 antibodies may or may not be pathogenic in and of themselves [1, 2, 49–51]. More recent articles also imply an interplay between innate and adaptive immunity [52, 53]. Affected organs display a striking resemblance on histologic examination [1, 43, 45], including the following: (a) dense lymphoplasmacytic infiltrate rich in IgG4^+^ plasma cells, typically >10/HPF and/or an IgG4^+^/IgG ratio >40%; (b) storiform fibrosis, resembling woven fibers or the spokes of a cartwheel; and (c) obliterative phlebitis [1, 43, 45, 54]. Tissue eosinophilia and elevated serum IgG4 > 135 mg/dL are also supportive of IgG4-RD [1, 43, 45, 54].

Comprehensive diagnostic criteria for IgG4-RD incorporating histology with clinical, serologic, and imaging features are now available. The first set of criteria came from content experts in Japan in 2011 [55] and was recently revised in 2020 [56]. Their approach uses the presence or absence of key disease features to assign the diagnosis as definite, probable, or possible. The second set of criteria comes from the American College of Rheumatology/European League Against Rheumatism in 2019 as the IgG4-RD Classification Criteria [57]. This latter approach is constructed in a stepwise fashion. Entry criteria and exclusion criteria must be reviewed before inclusion criteria are scored using a points-based system. Total inclusion points >20 are considered to meet the classification criteria for IgG4-RD. Both criteria are discussed in greater detail in these respective articles [55–57].

**Figure 3:**
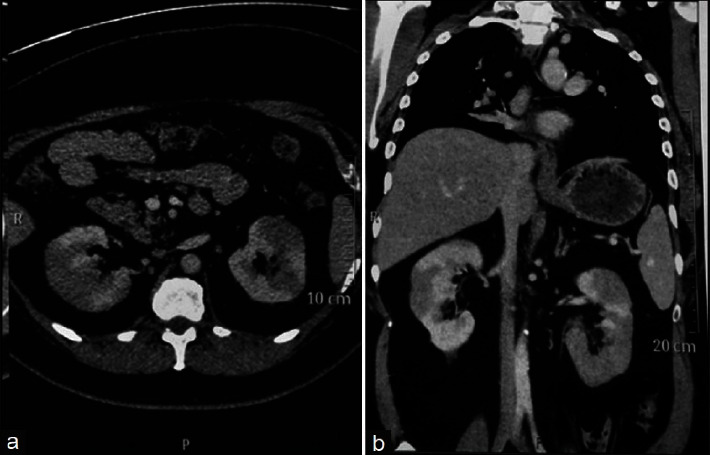
Contrast-enhanced computed tomography scan of the abdomen highlighting IgG4-related heterogenous enhancement of the kidneys with hypodensities at the lower pole. Adapted from Korivi et al. [62] as published in the *Indian Journal of Nephrology*. Reproduced under the Creative Commons Attribution-Noncommercial-Share Alike 3.0 Unported License.

In the kidney, IgG4-RD frequently presents a diagnostic challenge. Tubulointerstitial, glomerular, and perirenal involvements may all be noted and may mimic other autoimmune and neoplastic diseases. It is, therefore, imperative for the clinician and the pathologist to maintain appropriate clinical suspicion of IgG4-RD and its renal manifestations. Serologically, elevated serum IgG4 and IgE levels with hypocomplementemia are consistent with IgG4-RKD [43, 44]. Radiologically, one of the most reliable findings is the presence of multiple low-attenuation renal lesions on contrast-enhanced CT, as depicted in Figure 3. Additional findings may include diffuse kidney enlargement and solitary renal masses mimicking neoplasms, among others [43, 58]. Clinically, the onset and course of renal involvement can be acute but are generally slowly progressive [1, 59–61]. Key renal manifestations are individually reviewed in subsequent paragraphs.

## 3. Tubulointerstitial Involvement

To date, the most well-recognized renal manifestation of IgG4-RD is tubulointerstitial nephritis (IgG4-TIN) [34, 36, 48, 63]. Two key biopsy series provided initial insights into this entity. In 2010, Saeki and colleagues demonstrated in a Japanese cohort that IgG4-TIN was male predominant with a mean age of 65 and that 95% of patients had associated extra-renal IgG4-RD manifestations [34]. In 2011, Raissian and colleagues corroborated this in an American cohort [48]. Their findings demonstrated that IgG4-TIN was male predominant with a mean age of 65 and that 83% of patients had associated extra-renal IgG4-RD manifestations. Hence, it appears that the demographics of IgG4-TIN mimic those of IgG4-RD as a whole. It also appears that IgG4-TIN is rarely present in isolation without other organ manifestations. The subsequent literature has further supported these findings [64–66].

Interestingly, IgG4-TIN differs from other forms of TIN in several distinct ways. First, unlike drug-induced TIN, IgG4-TIN does not appear to be associated with urinary excretion of white blood cell casts [64]. Second, serum C-reactive protein tends to be normal [34], which is hypothesized to result from relatively indolent and low-grade autoimmune activity. Third, an elevated serum IgG4 concentration >135 mg/dL is observed in more than half of cases [43, 48]. Fourth, low-density renal lesions may be seen on enhanced CT [43, 48]. Fifth, and most importantly, histologic examination reveals unique tubulointerstitial features. These include storiform fibrosis plus prominent interstitial lymphoplasmacytic infiltrates with increased numbers of IgG4^+^ plasma cells, typically >10/HPF and/or an IgG4^+^/IgG ratio >40% [43]. The composition of cellular infiltrates may change as the disease progresses, with a mixture of lymphocytes and plasma cells in the early stages and attenuation of inflammatory cells with advanced fibrosis in the late stages [66]. Additional suggestive histologic features include the following: well-demarcated borders, cortical and medullary involvement with extension into and beyond the renal capsule, and the presence of plasma cell nests encased by fibrosis, creating a “bird's eye” pattern [43, 63, 66]. However, an abundance of IgG4^+^ plasma cells in renal tissue alone is not sufficiently specific, as it may also be seen in lupus, Sjogren syndrome, vasculitis (including ANCA-associated vasculitis and hypocomplementemic urticarial vasculitis), diabetic kidney disease, other forms of TIN, chronic pyelonephritis, and lymphoma, which need to be excluded with clinical, imaging, and serologic correlation [43, 44, 48, 67]. Histologic features against IgG4-TIN include necrotizing angiitis, granulomatous lesions, neutrophilic infiltration, and advanced tubulitis [43].

**Figure 4:**
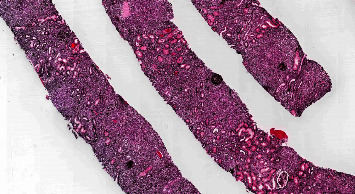
Marked tubulointerstitial effacement highlighted on silver stain in IgG4-RD. Reproduced with permission from PathologyOutlines.com.

In practice, clinical, serological, radiographic, and histological features must be considered together. Holistic diagnostic criteria for IgG4-TIN were proposed in 2011 by Raissian and colleagues [48]. Their criteria require the presence of histology compatible with IgG4-TIN plus at least one serologic, radiologic, or extra-renal manifestation suggestive of IgG4-RD [48]. An example of histologic IgG4-TIN is depicted in Figure 4.

## 4. Glomerular Involvement

Glomerular manifestations of IgG4-RD have also been observed, although to a much lesser degree than IgG4-TIN. The most common glomerular injury pattern is membranous glomerulonephritis, termed IgG4-related MGN. The prevalence of this entity is reported as approximately 7% (4/58) from two separate case series of patients with IgG4-TIN [34, 48]. In a key case series of IgG4-related MGN, patients typically presented with nephrotic range proteinuria and elevated serum creatinine, and 56% (5/9) of biopsies had overlapping TIN [68, 69]. A separate analysis of more than twenty case studies reported a similar 62% prevalence of IgG4-related MGN overlapping with TIN [70]. The pathogenesis of IgG4-related MGN is not fully understood but is likely distinct from the destructive inflammatory process in IgG4-RD involving other organs [71]. In primary MGN, IgG4 is typically the dominant IgG subclass identified on kidney biopsy by immunofluorescence with diffuse and global granular capillary wall staining, which correlates to subepithelial immune complex deposits on electron microscopy [72–74]. In contrast, MGN in the setting of IgG4-RD has multiple unique features. First, primary MGN is not associated with extra-renal IgG4-RD. Second, storiform fibrosis and lymphoplasmacytic tubulointerstitial inflammation are not typical histologic features of primary MGN. Third, almost all patients with MGN in the setting of IgG4-RD are negative for circulating anti-M type phospholipase A2 receptor (PLA2R) antibody by serology and for PLA2R staining in renal tissue, whereas PLA2R is the most common target antigen in primary MGN [70, 75]. As a direct result, serum PLA2R antibody positivity is an exclusion criterion in the ACR/EULAR 2019 IgG4-RD Classification Criteria [57]. Fourth, immunofluorescence for C1q typically shows negative to only segmental and weak granular glomerular capillary wall staining in primary MGN but can have diffuse and strong staining in some cases of MGN in IgG4-RD [70, 76]. Fifth, as compared to IgG4-TIN, IgG4-MGN is more likely to demonstrate an incomplete response to glucocorticoid administration [38, 68, 70]. Collectively, these unique patterns suggest that IgG4-MGN is likely an independent manifestation of IgG4-RD, distinct from primary MGN and distinct from IgG4-TIN, and likely represents an immune complex-mediated glomerulonephritis secondary to IgG4-RD. The nomenclature of IgG4-related MGN has been proposed [71].

**Figure 5:**
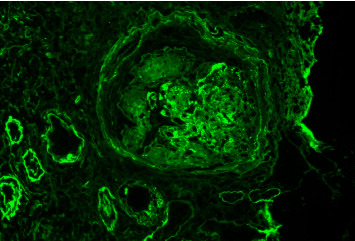
Granular IgG deposits within glomerular mesangial regions and capillary loops, as well as within tubular basement membranes and Bowman capsule. Background glomerulus shows features suggestive of diabetes. Reproduced with permission from PathologyOutlines.com.

Additional glomerular manifestations in IgG4-RD are very rare. Individual case reports have been published describing IgG4-RD associated with immune complex-mediated glomerulonephritis with various glomerular injury patterns, including membranoproliferative, mesangial proliferative, endocapillary proliferative, and crescentic patterns [34, 77–85]. There is also the potential for an association with diabetic glomerulosclerosis, as IgG4-related pancreatitis would predispose patients to diabetic kidney disease [64, 67].  Figure 5 depicts one example of glomerular disease in IgG4-RD on a background of diabetes. Further case reports and the collection of clinical, laboratory, radiologic, and histologic data are needed to better determine the nature of possible relationships between glomerular diseases and IgG4-RD (Appendix).

## 5. Additional Renal Involvement

IgG4-RD may also involve the renal pelvis, urinary tract, and retroperitoneum. In these instances, secondary renal dysfunction may occur due to obstructive hydronephrosis. IgG4-related pyelitis is the primary manifestation at the renal pelvis, which is typically detected radiographically as a pseudotumour and/or as renal pelvic wall thickening [86, 87]. Indeed, the presence of renal pelvic wall thickening is incorporated as an imaging criterion in the ACR/EULAR 2019 IgG4-RD Classification Criteria and in the Saeki and colleagues 2020 IgG4-RKD Proposed Criteria [44, 57]. More distally, ureteral IgG4-RD can manifest as inflammatory pseudotumours and segmental ureteritis [88, 89]. In the bladder, transmural bladder wall thickening and tumour-like masses have been documented [90–95]. Lymphoplasmacytic infiltration of the prostate gland may present as prostatitis and enlargement, as well as periprostatic tumour-like masses [96]. In the urethra, IgG4-related caruncles and mass lesions have been reported [97–99]. Perhaps most notably, retroperitoneal involvement is a well-described manifestation of IgG4-RD, and it is certainly plausible that many cases of presumed idiopathic retroperitoneal fibrosis (i.e., Ormond's disease) are actually secondary to IgG4-RD [41, 42, 100].

## 6. Treatment Considerations

Corticosteroids are the bedrock of initial therapy in IgG4-RD, irrespective of the presence or absence of renal involvement. A response to corticosteroids is usually prompt and favourable—a lack of response to steroids ought to evoke consideration of alternate diagnostic possibilities [65, 101–104]. Of note, corticosteroid regimens for IgG4-RD induction vary. A general approach involves oral prednisone at 0.6 mg/kg/day for approximately 4 weeks followed by a gradual taper either to zero or to a maintenance dose to sustain response [103]. Serum IgG4 levels, serum complement levels, imaging, and organ-specific bloodwork (e.g., creatinine) are repeated serially to assess the treatment response. Unfortunately, up to 30% of patients treated with glucocorticoids can experience relapse [102, 104]. In the setting of IgG4-RKD, there are case reports of patients requiring maintenance hemodialysis and renal transplantation despite glucocorticoid induction therapy [65, 105]. Hence, early diagnosis and treatment for patients with IgG4-RKD are essential to maximize the chances of renal recovery.

Indeed, repeated courses of glucocorticoids for reinduction therapy are undesirable due to the side effects. Hence, steroid-sparing agents, including azathioprine, mycophenolate, and rituximab, among others, have been used for longer-term maintenance therapy [103]. Rituximab therapy has the most efficacy data available to date [106, 107]. Whether a steroid-sparing agent should be initiated at the disease onset with glucocorticoids is controversial and remains a topic of debate [103]. In addition, procedural intervention may be required (e.g., ureteric stenting, nephrostomy tube insertion, and biliary stenting), depending on the sites and extent of involvement. In the setting of IgG4-RKD, renal replacement therapy may be necessary in rare cases. However, with treatment, renal function appears to improve readily, with sustained renal recovery on maintenance therapy [65].

## 7. Conclusion

Renal manifestations of IgG4-related disease (IgG4-RD) are rich and diverse, yet the disease is likely underdiagnosed. Delays in the diagnosis and treatment of renal manifestations of IgG4-RD can jeopardize the ability to obtain renal recovery. The primary objective of this review is to improve earlier recognition of IgG4-RD and, in particular, IgG4-related renal disease. We recommend that all clinicians who may be involved in the care of patients with IgG4-RD, particularly nephrologists, rheumatologists, general internists, and renal pathologists, consider the possibility of renal involvement in patients with known or suspected extra-renal IgG4-RD. We also recommend that clinicians consider IgG4-RD in the differential diagnosis of patients with newly identified renal masses and in patients with idiopathic retroperitoneal fibrosis. Awareness of renal manifestations of IgG4-RD may promote more accurate and timely diagnosis, which, in turn, may improve clinical outcomes for patients. Moving forward, further studies are required to increase our understanding of the epidemiology and pathophysiology of IgG4-RD and the role of different immunosuppressive regimens in the therapy of IgG4-related renal disease.

## Figures and Tables

**Table 1:** Diagnostic criteria for IgG4-related kidney disease (IgG4-RKD) 2020 [44].

(1) Presence of some kidney damage, as manifested by abnormal urinalysis or urine marker(s) or decreased kidney function with either the elevated serum IgG level, hypocomplementemia, or the elevated serum IgE level
(2) Abnormal renal radiologic findings:
(a) Multiple low-density lesions on enhanced computed tomography
(b) Diffuse kidney enlargement
(c) Hypovascular solitary mass in the kidney
(d) Hypertrophic lesion of the renal pelvic wall without irregularity of the renal pelvic surface
(3) Elevated serum IgG4 level (IgG4 ≥ 135 mg/dl)
(4) Histologic findings in the kidney
(a) Dense lymphoplasmacytic infiltration with infiltrating IgG4-positive plasma cells >10/high power field (HPF) and/or IgG4/IgG-positive plasma cells >40%
(b) Characteristic fibrosis surrounding nests of lymphocytes and/or plasma cells
(5) Extra-renal organ(s):
(a) Dense lymphoplasmacytic infiltration with infiltrating IgG4-positive plasma cells >10/HPF and IgG4/IgG-positive plasma cells >40% in extra-renal organ(s)
(b) Imaging or clinical findings in extra-renal organ(s): Existence of one of the following items:
(1) Bilateral lacrimal gland swelling
(2) Bilateral submandibular or parotid gland swelling
(3) Imaging findings compatible with type 1 autoimmune pancreatitis
(4) Imaging features of retroperitoneal fibrosis
Definite:
1 + 3 + 4a + 4b
2 + 3 + 4a + 4b
2 + 3 + 5a
1 + 3 + 4a + 5a or 5b
2 + 3 + 4a + 5b
Probable:
1 + 4a + 4b
2 + 4a + 4b
2 + 5a
2 + 3 + 5b
Possible:
1 + 3
2 + 3
1 + 4a
2 + 4a
2 + 5b
Appendix
(1) Clinically and histologically, exclusion of the following diseases should be considered: ANCA-associated vasculitis, multicentric Castleman's disease, malignant lymphoma, and extramedullary plasmacytoma.
(2) Radiologically, exclusion of the following diseases should be considered: Malignant lymphoma, urinary tract carcinoma, renal infarction, and pyelonephritis (rarely, granulomatosis with polyangiitis, sarcoidosis, and metastatic carcinoma)

## Data Availability

The data used to support the findings of this study are available from the corresponding author upon request. As this is solely a review article, no original data were collected.
